# P-2181. Post-Exposure Prophylaxis (PEP) of Respiratory Syncytial Virus (RSV) Infection After High-Inoculum RSV Human Challenge: Analysis of a Randomized Double-Blind, Placebo-Controlled Trial of EDP-323, an Oral, Non-Nucleoside Polymerase Inhibitor Antiviral

**DOI:** 10.1093/ofid/ofaf695.2344

**Published:** 2026-01-11

**Authors:** John DeVincenzo, Alaa Ahmad, Shijie Chen, Scott T Rottinghaus

**Affiliations:** Enanta Pharmaceuticals, Watertown, MA; Enanta Pharmaceuticals, Watertown, MA; Enanta Pharmaceuticals, Watertown, MA; Enanta Pharmaceuticals, Watertown, MA

## Abstract

**Background:**

RSV causes high secondary transmission rates in families, hospitals, nursing homes and other settings. Breakthrough RSV infections occur despite RSV vaccination or monoclonal antibody injection. Certain patients at great risk of severe RSV do not respond to RSV vaccination. No effective RSV treatments exist. EDP-323, a first in class, potent, oral, non-nucleoside small molecule RSV polymerase (L-protein) inhibitor rapidly reduces viral load and disease severity when treatment is started after an active RSV infection is identified^1^. The efficacy of RSV antivirals used for post-exposure prophylaxis (PEP) is unknown.

**Figure:**
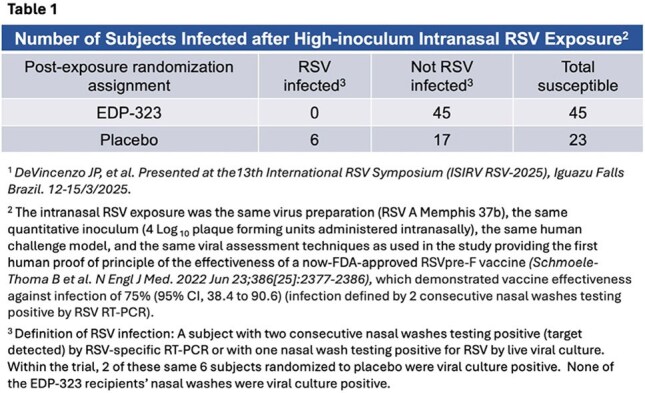

**Methods:**

A randomized, double-blind, placebo (PBO)-controlled study (NCT06170242) evaluated the efficacy, antiviral activity and safety, of EDP-323. Healthy volunteers received large intranasal exposures of a low-passage clinical strain of RSV-A (Memphis-37, 4 Log_10_

plaque forming units)^2^ on Day (D)0. RSV RT-PCR was performed on nasal washes collected twice daily on D2-12. If PCR-confirmed RSV infection had not occurred by D5am after RSV exposure, participants were randomized to receive daily oral EDP-323 high dose (600mg), low dose (200mg with 600mg loading dose) or PBO for 5D. PEP efficacy was evaluated in this pre-specified population using Fisher’s Exact test (two-tailed) of RSV-uninfected vs infected (pre-defined as PCR-positive on 2 consecutive specimens).

**Results:**

In this population, 68 RSV-exposed, susceptible subjects were randomized to receive EDP-323 (low dose N=24, high dose N=21) and 23 PBO. A 2x2 table shows infection outcomes (Table 1). 26% (6/23) of PBO recipients became infected vs 0% (0/45) of EDP-323 recipients (*P*< 0.001). Evaluated separately, the 2 EDP-323 dosing groups’ PEP effects were statistically significant (low dose

*P*= 0.009, high dose *P*= 0.022) vs PBO. No serious TEAEs, severe AEs, or AEs leading to treatment discontinuation or study withdrawal occurred. Frequencies of treatment-emergent adverse events (TEAEs) were similar across EDP-323 and PBO groups.

**Conclusion:**

EDP-323 appears highly effective in preventing RSV infections when initiated up through 5 days after high-inoculum intranasal RSV exposure. These findings support further evaluation of EDP-323 for prophylaxis.

**Disclosures:**

John DeVincenzo, MD, Enanta Pharmaceuticals: Employee of Enanta Pharmaceuticals|Enanta Pharmaceuticals: Stocks/Bonds (Public Company) Alaa Ahmad, PhD, Enanta Pharmaceuticals: Employee of Enanta Pharmaceuticals|Enanta Pharmaceuticals: Stocks/Bonds (Public Company) Shijie Chen, PhD, Enanta Pharmaceuticals: Employee of Enanta Pharmaceuticals|Enanta Pharmaceuticals: Stocks/Bonds (Public Company) Scott T Rottinghaus, MD, Enanta Pharmaceuticals: Employee of Enanta Pharmaceuticals|Enanta Pharmaceuticals: Stocks/Bonds (Public Company)

